# Prospective Estimation of the Prevalence of Thromboembolism in Dogs With Inflammatory Protein‐Losing Enteropathy

**DOI:** 10.1111/jvim.70098

**Published:** 2025-04-12

**Authors:** Nene Oishi, Hiroshi Ohta, Masahiro Tamura, Kiwamu Hanazono, Kenjiro Miyoshi, Nozomu Yokoyama, Genya Shinbo

**Affiliations:** ^1^ Companion Animal Internal Medicine, Department of Companion Animal Clinical Sciences, School of Veterinary Medicine Rakuno Gakuen University Ebetsu Japan; ^2^ Laboratory of Veterinary Internal Medicine, Graduate School of Veterinary Medicine Hokkaido University Sapporo Japan; ^3^ Veterinary Diagnostic Imaging, Department of Companion Animal Clinical Sciences, School of Veterinary Medicine Rakuno Gakuen University Ebetsu Japan; ^4^ Veterinary Teaching Hospital, Graduate School of Veterinary Medicine Hokkaido University Sapporo Japan

**Keywords:** canine, computed tomography angiography, protein‐losing enteropathy, thrombosis

## Abstract

**Background:**

Inflammatory protein‐losing enteropathy (iPLE) is thought to be associated with a hypercoagulable state and may predispose dogs to thromboembolism (TE). However, little information is available regarding the prevalence of TE in dogs with iPLE.

**Objectives:**

Estimate the prevalence of TE in dogs with iPLE and collect clinical and clinicopathologic data from dogs with iPLE with and without TE.

**Animals:**

Twenty‐two client‐owned dogs with iPLE.

**Methods:**

Prospective, descriptive study. Dogs definitively diagnosed with iPLE based on standard diagnostic criteria and histopathology were recruited between January 2019 and February 2024. At the time of gastrointestinal endoscopic examination, dogs with iPLE underwent thoracic and abdominal computed tomography angiography to detect TE. Clinical (e.g., clinical severity, use of corticosteroids) and clinicopathologic (e.g., albumin concentration, coagulation parameters) data were collected from dogs with iPLE with and without TE.

**Results:**

Thromboembolism was found in 3/22 dogs (13.6%, 95% confidence interval: 2.9–34.9) with iPLE. The three dogs with iPLE and TE had thrombi in the left external iliac artery, pulmonary artery of the right caudal lobe, and main portal vein, respectively. The dogs with thrombi in the left external iliac artery or pulmonary artery did not show any clinical signs associated with TE, whereas the dog with portal vein thrombosis had transudative peritoneal effusion.

**Conclusion and Clinical Importance:**

We estimated the prevalence of TE in dogs with iPLE. In dogs with iPLE, TE could be underestimated because some affected dogs have subclinical TE.

**Abbreviations**
ALB: albumin
AT: antithrombin
CCECAI: canine chronic enteropathy clinical activity index
CIBDAI: canine inflammatory bowel disease activity index
CIE: chronic inflammatory enteropathy
CT: computed tomography
IBD: inflammatory bowel disease
iPLE: inflammatory protein‐losing enteropathy
PLE: protein‐losing enteropathy
PTE: pulmonary thromboembolism
RI: Reference interval
TE: thromboembolism
WSAVA: World Small Animal Veterinary Association

## 1 Introduction

Protein‐losing enteropathy (PLE) in dogs is a syndrome characterized by abnormal loss of proteins through the gastrointestinal tract [1], resulting in hypoalbuminemia. In dogs, PLE is observed in chronic small intestinal diseases, such as primary lymphangiectasia, chronic inflammatory enteropathy (CIE), and intestinal lymphoma [2, 3]. It can be caused by abnormal lymphatic structure or function but can also be caused by severe inflammation within the gastrointestinal tract [1, 4, 5]. Thus, for the purpose of our study, the term inflammatory PLE (iPLE) is used as reported previously [6]. Clinical signs of iPLE include small intestinal diarrhea, vomiting, anorexia, weight loss, and systemic manifestations of severe hypoalbuminemia, such as ascites, pleural effusion, and peripheral edema. In addition, dogs with iPLE reportedly experience several systemic complications, such as hypocobalaminemia [7, 8], ionized hypocalcemia [2, 9], hypovitaminosis D [10, 11], and thromboembolism (TE) [12, 13, 14].

Dogs with iPLE are suggested to be at increased risk of TE, and thromboembolic complications are thought to result from underlying hypercoagulability [1, 12, 13]. Although information about the prevalence of TE in dogs with iPLE is limited, a previous article that summarized the results of multiple studies reported that the overall frequency of TE in dogs with PLE was 2.6% (46/1798) [14]. However, the incidence of TE might be underestimated in veterinary patients because of the limited availability of diagnostic imaging that allows an antemortem diagnosis, and a limited number of cases submitted for necropsy. Thus, the true prevalence of TE in dogs with iPLE is still unknown.

In humans, inflammatory bowel disease (IBD) is a well‐established independent risk factor for TE, and patients with IBD commonly experience venous thromboembolic events, such as deep vein thrombosis and pulmonary TE (PTE) [15]. The pathogenesis of TE in humans with IBD is incompletely understood and thought to be multifactorial. It is hypothesized that chronic inflammation triggers a procoagulant state that leads to endothelial dysfunction, thrombocytosis, platelet hyperaggregation, hyperfibrinogenemia, and impaired fibrinolysis [15, 16, 17]. Additional risk factors for venous TE in patients with IBD include major abdominal surgery, long‐term placement of central venous catheters, and treatment with corticosteroids [18]. In dogs with iPLE, the exact mechanism of TE has not been clarified, and the etiology is thought to be multifactorial as in humans with IBD, although there are many differences in the etiology and pathophysiology between dogs with iPLE and humans with IBD. A previous study that investigated the relationship between a hypercoagulable state as measured by thromboelastography and conventional coagulation tests in dogs with iPLE found that the hypercoagulable state cannot be solely attributed to any specific coagulation abnormality, including decreased antithrombin (AT) activity [19].

Our aim was to estimate the prevalence of TE in client‐owned dogs with iPLE. A secondary aim was to collect clinical and clinicopathologic data from dogs with iPLE with and without TE.

## 2 Materials and Methods

### 2.1 Study Design

Ours was a prospective descriptive study. Client‐owned dogs presented for investigation of chronic gastrointestinal signs with a duration of ≥ 3 weeks and hypoalbuminemia were prospectively enrolled between January 2019 and February 2024 at two referral veterinary teaching hospitals at the time gastrointestinal endoscopy was performed to obtain gastrointestinal biopsy specimens.

### 2.2 Cases

Dogs suspected to have iPLE that underwent upper gastrointestinal endoscopy with or without lower intestinal endoscopy concurrently with computed tomography (CT) angiography at Hokkaido University Veterinary Teaching Hospital from January 2019 to January 2020 or at Rakuno Gakuen University Animal Medical Center from May 2020 to February 2024 were included. Inclusion criteria for dogs with iPLE were as follows: hypoalbuminemia (< 2.6 g/dL), histological evidence of gastrointestinal tract inflammatory diseases known to be associated with PLE (e.g., lymphocytic‐plasmacytic enteritis) in endoscopic biopsy specimens, and the absence of other causes of chronic gastrointestinal signs and hypoalbuminemia based on physical examination, CBC, serum biochemistry, fecal examination, urinalysis, radiography, and abdominal ultrasonography. In all dogs, albuminuria and hepatic failure were excluded based on urine protein‐to‐creatinine ratio and serum bile acid concentration, respectively. Exocrine pancreatic insufficiency was excluded by verifying that serum trypsin‐like immunoreactivity was > 5 ng/mL in two dogs, and hypoadrenocorticism was excluded by determining that basal serum cortisol concentration was > 2 μg/dL or a normal response to ACTH stimulation in four dogs. Dogs that had already received treatment (e.g., glucocorticoids, dietary intervention, antithrombotic drugs) before gastrointestinal endoscopy and CT angiography were included. Dogs with large cell or small cell gastrointestinal lymphoma diagnosed by histological examination of endoscopic biopsy specimens were excluded. The study was approved by the Ethics Screening Committees of Hokkaido University Veterinary Teaching Hospital (permission number CR‐24‐02) and Rakuno Gakuen University (permission number 2023‐05). All owners provided informed consent for the inclusion of their dogs in the study.

### 2.3 Histopathology

For histopathological examination, biopsy specimens were obtained from the stomach, proximal duodenum, distal duodenum, and ileum. During endoscopy, at least six samples were collected from each previously noted segment of the gastrointestinal tract. Histopathological examination was conducted by an American College of Veterinary Pathology board‐certified pathologist (Y. K.) using a scoring system based on World Small Animal Veterinary Association (WSAVA) guidelines [20].

### 2.4 Computed Tomography Angiography

Immediately before gastrointestinal endoscopy, thoracic and abdominal CT angiography was performed under general anesthesia. The region from the thoracic inlet to the anus was examined.

An 80‐slice CT machine (Aquillion PRIME, Canon Medical Systems, Tochigi, Japan) was used to perform CT angiography at Hokkaido University Veterinary Teaching Hospital. Helical images were acquired with dogs in sternal recumbency. For contrast‐enhanced CT angiography, iopamidol (Iopamidol injection 150, Teva Takeda Pharma Ltd. Nagoya, Japan) or iohexol (Iohexol injection 300, Hikari Pharmaceutical Co. Ltd. Tokyo, Japan) was used as a contrast medium and administered at a dose of 600 mg I/kg IV in the cephalic vein using a power injector (Dual Shot GX7, Nemoto Kyorindo Co. Ltd. Tokyo, Japan). For all dogs, the injection duration was set at 20 s. Computer software determined the timing of the arterial, venous, and delayed phases based on the time the contrast medium appeared in the abdominal aorta. Arterial phase images were acquired 15 s after the contrast medium appeared in the abdominal aorta. Venous phase images were acquired 20 s after the arterial phase. Delayed phase images were acquired 180 s after the contrast medium was injected.

A 16‐slice CT machine (Bright Speed Elite SD, GE Health Care, Tokyo, Japan) was used to perform CT angiography at Rakuno Gakuen University Animal Medical Center. Helical images were acquired with dogs in sternal recumbency. For contrast‐enhanced CT angiography, iohexol (Omnipaque 300, GE Healthcare, Oslo, Norway) was used as a contrast medium and was administered at a dose of 600 mgI/kg IV in the cephalic vein using a power injector (Stellant CT injection system, Bayer Yakuhin Ltd. Osaka, Japan). For all dogs, the injection duration was set at 20 s. Computer software determined the timing of the arterial, venous, and delayed phases based on the time the contrast medium appeared in the abdominal aorta. Arterial, venous, and delayed phase images were acquired at 11, 60, and 180 s, respectively, after the contrast medium appeared in the abdominal aorta.

### 2.5 Image Evaluation

At Hokkaido University Veterinary Teaching Hospital, CT angiography images were reviewed by a member of the faculty of veterinary diagnostic imaging (G. S.). At Rakuno Gakuen University Animal Medical Center, CT angiography images were reviewed by members of the faculty of veterinary diagnostic imaging (K. H. or K. M.). Angiography images were reviewed for the following data: presence or absence of thrombus, number of thrombi, and location of thrombi. Multiplanar reconstructions were used when necessary for further evaluation. The presence of a thrombus was defined as the identification of a filling defect in the atrial, venous, and delayed phases.

### 2.6 Data Collection

The following information was collected from the medical records at the time of gastrointestinal endoscopy: breed, age, weight, sex, clinical signs, previous treatment, platelet count, plasma concentrations of albumin (ALB), globulin, and C‐reactive protein; prothrombin time; activated partial thromboplastin time; fibrinogen concentration; D‐dimer concentration; and, AT activity. D‐dimer concentrations were represented based on the fold change with respect to the upper limit of the corresponding reference interval (i.e., plasma D‐dimer concentration/upper limit of the reference interval) because the upper limit of the reference interval of D‐dimer concentration differed between the two veterinary teaching hospitals. In addition, the histological score (WSAVA score) was determined after histological diagnosis. The severity of clinical signs was evaluated by using two previously described activity indexes: the canine inflammatory bowel disease activity index (CIBDAI) [21] and the canine chronic enteropathy clinical activity index (CCECAI) [7].

## 3 Results

### 3.1 Cases

During the study period, 28 dogs with hypoalbuminemia were suspected to have PLE and underwent gastrointestinal endoscopy and CT angiography. On the basis of histological evaluation, 22 dogs were diagnosed with iPLE and included in the study. Mucosal biopsy specimens were obtained by endoscopy from the stomach, proximal duodenum, and distal duodenum in all 22 dogs, and from the ileum in 14 dogs. We could not obtain ileal biopsy specimens from eight dogs. Of these 22 dogs with iPLE, 20 dogs were diagnosed with lymphocytic‐plasmacytic enteritis with lymphangiectasia, one dog was diagnosed with eosinophilic enteritis with lymphangiectasia, and one dog was diagnosed with neutrophilic enteritis with lymphangiectasia. Four dogs diagnosed with small cell lymphoma and two dogs diagnosed with large cell lymphoma were excluded from the study. The median age and median body weight of the 22 dogs with iPLE were 113 months (range, 65–195 months) and 6.1 kg (range, 2.8–13.7 kg), respectively. The dogs with iPLE consisted of 11 females (9 spayed females and 2 intact females) and 11 males (10 castrated males and 1 intact male). The breeds of the dogs with iPLE were as follows: Shiba Inu (*n* = 5), Toy Poodle (*n* = 4), Miniature Dachshund (*n* = 3), Pomeranian (*n* = 2), Miniature Schnauzer (*n* = 1), Pug (*n* = 1), Chihuahua (*n* = 1), Italian Greyhound (*n* = 1), Kooikerhondje (*n* = 1), Shih Tzu (*n* = 1), Beagle (*n* = 1), and Yorkshire Terrier (*n* = 1).

### 3.2 Computed Tomography Angiography

**FIGURE 1:**
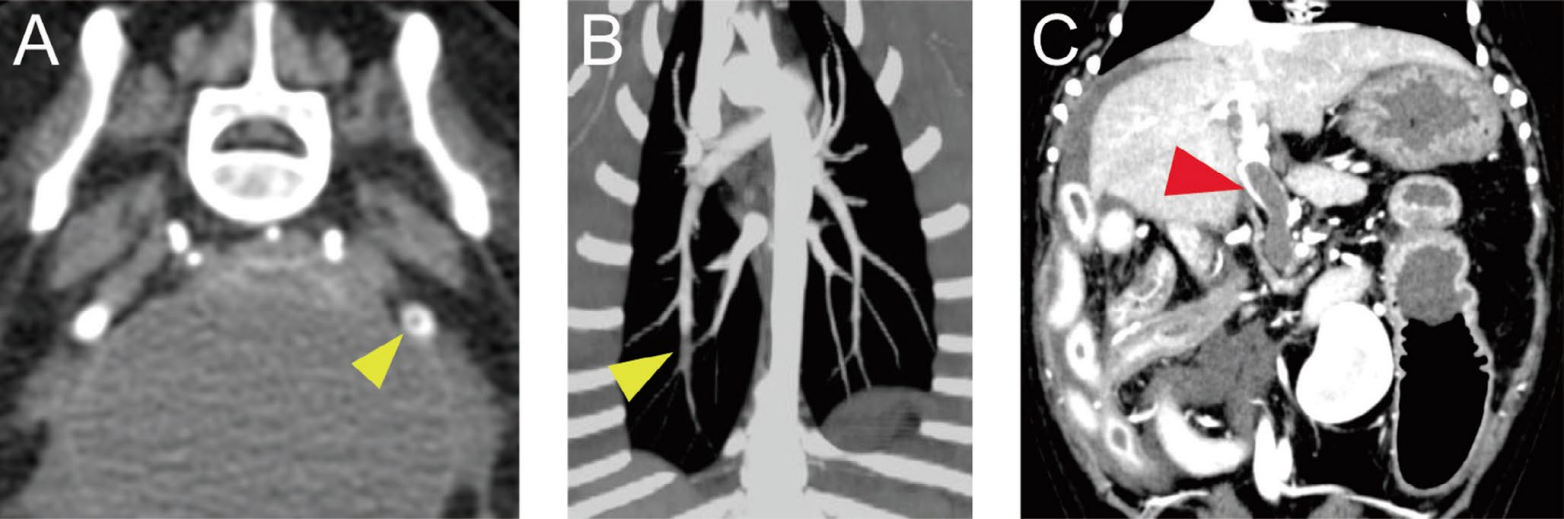
Computed tomography angiography images of the thrombus in three dogs with inflammatory protein‐losing enteropathy with thromboembolism. (A) Transverse arterial phase image of left external iliac artery thrombosis (arrowhead) in a Miniature Dachshund, (B) dorsal maximum intensity projection arterial phase image of pulmonary artery thrombosis of the right caudal lobe (arrowhead) in a Miniature Schnauzer, and (C) dorsal aspect of a venous phase image of main portal vein thrombosis (arrowhead) in a Pug.

Three of the 22 dogs with iPLE (13.6%, 95% confidence interval [CI]: 2.9–34.9) had TE. In these three dogs, thrombi were found in the left external iliac artery, pulmonary artery of the right caudal lung lobe, and main portal vein, respectively (Figure 1).

### 3.3 Clinical and Clinicopathological Findings in Dogs With Inflammatory Protein‐Losing Enteropathy With and Without Thromboembolism

The clinical findings and prior treatments for dogs with iPLE with and without TE are summarized in Table 1. The median CIBDAI scores of dogs with and without TE were six (range, 1–15) and 11 (range, 0–16), respectively. The median CCECAI scores of dogs with and without TE were eight (range, 1–18) and 6 (range, 0–16), respectively. The median WSAVA score of dogs with and without TE was six (range, 3–7) and 5 (range, 2–13), respectively.

**TABLE 1:** Clinical findings and prior treatments in dogs with inflammatory protein‐losing enteropathy with and without thromboembolism.

Variable	Thromboembolism (*n* = 3)	Without thromboembolism (*n* = 19)
Age (month), median (range)	124 (111–132)	111 (65–195)
Weight (kg), median (range)	6.5 (6.3–6.7)	6.1 (2.8–11.5)
Female, number (%)	1 (33.3)	10 (52.6)
CIBDAI, median (range)	6 (1–15)	11 (0–16)
CCECAI, median (range)	8 (1–18)	6 (0–16)
WSAVA score, median(range)	6 (3–7)	5 (2–13)
Lacteal dilation score, median (range)	1 (1–3)	1 (1–3)
Prednisolone administration, number (%)	3 (100)	12 (63.2)
Chlorambucil administration, number (%)	0 (0)	1 (5.3)
Antibiotics administration, number (%)	1 (33.3)	7 (36.8)
Clopidogrel administration, number (%)	1 (33.3)	4 (21.1)
Rivaroxaban administration, number (%)	0 (0)	1 (5.3)
Ultra‐low‐fat diet (%)	0 (0)	3 (15.8)
Low‐fat dry diet (%)	1 (33.3)	6 (31.6)
Ultra‐low‐fat diet and low‐fat dry diet (%)	0 (0)	3 (15.8)
Hydrolyzed dry diet (%)	2 (66.7)	0 (0)

Abbreviations: CCECAI, canine chronic enteropathy clinical activity index; CIBDAI, canine inflammatory bowel disease activity index; WSAVA, world small animal veterinary association.

Eighteen dogs with iPLE received at least one of the following treatments before CT and endoscopic examination. Dogs with iPLE were treated with a combination of prednisolone (0.5 to 3.0 mg/kg/day; 15/22), chlorambucil (2 mg/m^2^/day; 1/22), antibiotics (8/22), clopidogrel (1.2 to 3.5 mg/kg/day; 5/22), rivaroxaban (1.1 mg/kg/day; 1/22), an ultra‐low‐fat diet (3/22), a low‐fat dry diet (7/22), a combination of an ultra‐low‐fat diet and a low‐fat dry diet (3/22), and a hydrolyzed dry diet (2/22).

Dogs with iPLE with TE were treated with prednisolone (0.6 to 1.5 mg/kg/day; 3/3), antibiotics (1/3), clopidogrel (1.2 mg/kg/day for 14 days; 1/3), a low‐fat dry diet (17 days; 1/3), and a hydrolyzed dry diet (2/3) before CT and endoscopic examination. The median duration of prednisolone administration in the three dogs with iPLE with TE was 4 days (range, 4–9 days). The durations of the hydrolyzed dry diet in the two dogs with iPLE with TE were 4 days and > 5 years, respectively.

Dogs with iPLE without TE were treated with prednisolone (0.5 to 3.0 mg/kg/day; 12/19), chlorambucil (2 mg/m^2^/day; 1/19), antibiotics (7/19), clopidogrel (1.9 to 3.5 mg/kg/day; 4/19), rivaroxaban (1.1 mg/kg/day for 1 day; 1/19), an ultra‐low‐fat diet (3/19), a low‐fat dry diet (6/19), and a combination of an ultra‐low‐fat diet and a low‐fat dry diet (3/19) before CT and endoscopic examination. In dogs with iPLE without TE, the median duration of prednisolone administration was 5 days (range, 2–84 days) in seven dogs and unknown in five dogs. The median duration of clopidogrel administration was 36 days (range, 27–45 days) in four dogs with iPLE without TE. The median duration of an ultra‐low‐fat diet was 11 days (range, 7–168 days) in three dogs with iPLE without TE. The median duration of a low‐fat dry diet was 9 days (range, 7–210 days) in three dogs with iPLE without TE, and was unknown in three dogs with iPLE without TE. The duration of a combination of an ultra‐low‐fat diet and a low‐fat dry diet was 7 days in one dog with iPLE without TE, and unknown in two dogs with iPLE without TE.

The clinicopathological results from dogs with iPLE with and without TE are summarized in Table 2. The proportion of dogs with iPLE with and without TE in which plasma globulin concentration was below the reference interval (RI) was 1/3 (33.3%) and 3/19 (15.8%), respectively. The proportion of dogs with iPLE with and without TE in which plasma CRP concentration was above the RI was 0/3 (0%) and 7/19 (36.8%), respectively. The proportion of dogs with iPLE with and without TE in which platelet count was above the RI was 1/3 (33.3%) and 5/19 (26.3%), respectively. The proportion of dogs with iPLE with and without TE in which prothrombin time was above the RI was 0/3 (0%) and 1/19 (5.3%), respectively. The proportion of dogs with iPLE with and without TE in which activated partial thromboplastin time was above the RI was 2/3 (66.7%) and 12/19 (63.2%), respectively. The proportion of dogs with iPLE with and without TE in which fibrinogen concentration was above the RI was 0/3 (0%) and 2/19 (10.5%), respectively. The proportion of dogs with iPLE with and without TE in which adjusted D‐dimer concentration was above the RI was 2/3 (66.7%) and 9/19 (47.4%), respectively. The proportion of dogs with iPLE with and without TE in which AT activity was below the RI was 3/3 (100%) and 15/19 (78.9%), respectively.

**TABLE 2:** Clinicopathological variables from dogs with inflammatory protein‐losing enteropathy with and without thromboembolism.

Variable	With TE (*n* = 3)				Without TE (*n* = 19)				
	Median (range)	Below RI (%)	Above RI (%)	Within RI (%)	Median (range)	Below RI (%)	Above RI (%)	Within RI (%)	RI
ALB (g/dL)	1.8 (1.5–2.3)	100	0	0	2.1 (1.2–2.5)	100	0	0	2.6–4.0
GLB (g/dL)	1.6 (1.1–2.1)	33.3	0	66.7	2.3 (1.3–4.3)	15.8	5.3	78.9	1.6–3.7
CRP (mg/dL)	0.3 (0–0.6)	NA	0	100	0.4 (0–11.7)	NA	36.8	63.2	< 0.7
Platelet count (×10^3^/μL)	340 (112–897)	33.3	33.3	33.3	498 (181–1090)	10.5	26.3	63.2	200–400
PT (s)	7.3 (7.2–8.4)	NA	0	100	7.7 (7.2–8.6)	NA	5.3	94.7	≤ 8.4
aPTT (s)	25.7 (19.7–28.4)	NA	66.7	33.3	20.5 (11.7–45)	NA	63.2	36.8	≤ 25.6
Fibrinogen concentration (mg/dL)	205 (136–233)	0	0	100	244 (150–536)	0	10.5	89.5	113–385
Adjusted D‐dimer	2.8 (0.5–22.2)	NA	66.7	33.3	0.97 (0.38–12.1)	NA	47.4	52.6	< 1
AT activity (%)	53 (32–93)	100	0	0	98 (63–158)	78.9	0	21.1	114–179

Abbreviations: ALB, plasma albumin concentration; aPTT, activated partial thromboplastin time; AT, antithrombin; CRP, C‐reactive protein; GLB, plasma globulin concentration; NA, not applicable; PT, prothrombin time; RI, reference interval; TE, Thromboembolism.

### 3.4 Dogs With Inflammatory Protein‐Losing Enteropathy With Thromboembolism

The dog with TE in the left external iliac artery was a Miniature Dachshund with an AT activity of 32%, adjusted D‐dimer fold change of 2.75, and serum ALB concentration of 1.8 g/dL. The dog with TE in the pulmonary artery of the right caudal lung lobe was a Miniature Schnauzer with an AT activity of 93%, adjusted D‐dimer fold change of 0.5, and serum ALB concentration of 2.3 g/dL. The dog with TE in the main portal vein was a Pug with an AT activity of 53%, adjusted D‐dimer fold change of 22.2, and serum ALB concentration of 1.5 g/dL. The dogs with TE in the left external iliac artery or in the pulmonary artery did not show any clinical signs associated with TE, whereas the dog with portal vein thrombosis had transudative peritoneal effusion.

## 4 Discussion

We prospectively investigated the prevalence of TE in dogs with iPLE using CT angiography and found that TE was detected in three of the 22 dogs with iPLE. We also found that two of the three dogs with iPLE with TE had subclinical TE.

The estimated prevalence of TE in dogs with iPLE was 13.6% in our study. However, only three of the 22 dogs with iPLE had TE, and the 95% CI of the estimated prevalence of TE was relatively wide. The number of dogs with iPLE, especially dogs with iPLE with TE, was small, leading to a crude estimation of the prevalence of TE in dogs with iPLE, which is a major limitation of our study. Thus, additional studies with larger numbers of cases are needed to more accurately estimate the prevalence of TE in dogs with iPLE.

In our study, two of three dogs with iPLE with TE did not show obvious clinical signs related to TE. In these two dogs, only small thrombi were found in the left external iliac artery and the pulmonary artery of the right caudal lung lobe, respectively. We prospectively investigated TE in dogs with iPLE using CT angiography, which enabled us to detect subclinical TE. Previous studies have reported the usefulness of CT angiography for the definitive diagnosis of portal vein thrombosis, and reported that CT is superior to abdominal ultrasonography in detecting portal vein thrombosis in dogs [22, 23]. In addition, PTE was reported to be the most common location of thrombus formation in dogs with PLE [13]. Thus, CT angiography is a useful screening modality for the diagnosis of TE in dogs with iPLE. In our study, CT angiographic examination included the thoracic and abdominal regions but did not include the entire body. Thus, it is possible that TE in areas other than the thoracic or abdominal regions was missed, potentially leading to an underestimation of the prevalence of TE. In addition, the CT images of each patient were evaluated by one of the three radiologists, and we did not evaluate the agreement in the interpretation of the CT images among the three radiologists. Therefore, future correction of these limitations of our study may lead to a more accurate estimation of the prevalence of TE in dogs with iPLE.

As mentioned previously, thrombocytosis and hyperfibrinogenemia secondary to chronic inflammation are thought to be risk factors of TE in humans with IBD [15, 16, 17]. In our study, thrombocytosis was found in only one dog with iPLE with TE, and increased fibrinogen concentration was not found in any dog with iPLE with TE. These results indicate that thrombocytosis and hyperfibrinogenemia do not seem to be independent risk factors of TE in dogs with iPLE.

In humans, decreased plasma AT concentrations are reported to be associated with increased risk of venous TE, and measurement of the plasma AT concentration should be considered for assessment of the individual risk of venous TE [24]. In dogs with iPLE, it is unknown whether decreased plasma AT activity is a contributory factor for TE. A previous study reported that the AT activity alone cannot predict the hypercoagulable state in dogs with PLE [19]. In our study, 3/3 dogs with iPLE with TE and 15/19 dogs with iPLE without TE had AT activity below the RI, respectively. Although all three dogs with iPLE with TE had decreased AT activity, substantial overlap in AT activity was observed between dogs with iPLE with TE and without TE. Our results indicated that the mechanism of TE in dogs with iPLE may be multifactorial, and not simply a consequence of decreased AT activity.

In humans, D‐dimer concentration is recognized as the most useful laboratory marker for the exclusion of venous thrombosis and PTE [25]. In dogs, a higher plasma D‐dimer concentration is reportedly more likely associated with TE, whereas a low D‐dimer concentration might be useful for the exclusion of TE [26, 27]. In our study, one dog with iPLE with subclinical TE in the pulmonary artery had an adjusted D‐dimer concentration within RI. In dogs with experimentally induced PTE, D‐dimer concentrations reach their peak within 2 h of the induction of PTE and return to baseline within 24 to 48 h [28]. Thus, it is possible that a thrombus might have formed > 48 h before the CT examination, and the D‐dimer concentration might have returned to within the normal reference range by the time of testing.

Our study did not exclude dogs with iPLE that had received prior treatment, such as glucocorticoids, dietary interventions, or antithrombotic drugs. In fact, 3/3 dogs with iPLE with TE and 12/19 dogs with iPLE without TE had received prednisolone previously. Exogenous and endogenous glucocorticoid excess is thought to be associated with increased risk of TE in humans and dogs. Previous studies have reported that administration of exogenous corticosteroids to healthy dogs or presence of endogenous glucocorticoid excess in dogs with hyperadrenocorticism results in a hypercoagulable state [29, 30]. In addition, 1/3 dogs with iPLE with TE and 5/19 dogs with iPLE without TE had received antithrombotic drugs previously. Thus, the effects of prior treatment on TE development or prevention could not be excluded.

Our study had an additional limitation. We could not obtain ileal biopsy specimens in eight dogs with iPLE. Thus, the possibility of gastrointestinal lymphoma in the ileum could not be excluded in these eight dogs with iPLE.

In conclusion, we estimated the prevalence of TE in a limited number of dogs with iPLE and suggest that TE could be underestimated because some dogs with iPLE may have subclinical TE.

## Disclosure

Authors declare no off‐label use of antimicrobials.

## Ethics Statement

This study was approved by the Ethics screening committee in Hokkaido University Veterinary Teaching Hospital (permission number CR‐24‐02) and in Rakuno Gakuen University (permission number 2023‐05). Informed owner consent was obtained in all cases. Authors declare that human ethics approval was not needed.

## Conflicts of Interest

The authors declare no conflicts of interest.
